# Clinical outcomes and opportunities for improvement across modified trauma quality improvement cohorts: a registry-based cohort study

**DOI:** 10.1186/s13049-026-01674-6

**Published:** 2026-08-17

**Authors:** Jonatan Attergrim, Lovisa Strömmer, Olof Brattström, Martin Jacobsson, Josef Al-Khalili, Elin Sun Cao, Kelvin Szolnoky, Johanna Berg, Martin Gerdin Wärnberg

**Affiliations:** 1 Department of Global Public Health, Karolinska Institutet, Stockholm, 171 77 Sweden; 2 Perioperative Medicine and Intensive Care, Karolinska University Hospital, Solna, Stockholm Sweden; 3 Department of Clinical Science, Intervention and Technology, Division of Surgery and Oncology, Karolinska Institutet, Karolinska University Hospital Huddinge, Stockholm, Sweden; 4 Department of Anesthesiology, Mora Hospital, Mora, Sweden; 5 Department of Biomedical Engineering and Health Systems, KTH Royal Institute of Technology, Huddinge, Sweden; 6 Department of Medical Epidemiology and Biostatistics, Karolinska Institutet, Stockholm, Sweden; 7 Department of Internal and Emergency Medicine, Skånes universitetssjukhus Malmö, Malmö, Sweden

**Keywords:** Trauma, Quality improvement, TQIP, Mortality, Glasgow Outcome Scale, Opportunities for improvement, Peer review, Registry-based cohort study

## Abstract

**Background:**

The American College of Surgeons Trauma Quality Improvement Program (TQIP) relies primarily on risk-adjusted mortality benchmarking, yet mortality alone may overlook non-fatal morbidity and care-process failures, particularly in cohorts where outcomes are driven by injury severity. This study aimed to analyse and compare 30-day mortality, cause of death, functional outcomes as measured by the Glasgow Outcome Scale (GOS), and opportunities for improvement (OFIs) identified through peer review across modified TQIP cohorts.

**Methods:**

Registry-based cohort study of 8,298 trauma patients at Karolinska University Hospital (2013–2023), classified into four modified TQIP cohorts: isolated severe TBI, blunt multisystem with TBI, blunt multisystem without TBI, and penetrating truncal injury. Remaining patients, of lower severity on average, formed a non-TQIP reference cohort. Cohort associations with mortality, unfavourable GOS, and OFI were assessed using cumulative sequential logistic regression (reference: non-TQIP cohort), progressively adjusting for patient characteristics, physiological status, and care processes, pooled across 20 imputed datasets. Cause of death, GOS levels, and OFI categories were compared using multinomial logistic regression.

**Results:**

All TQIP cohorts had elevated odds of mortality and unfavourable GOS. The blunt multisystem cohorts had the highest OFI rates (10.6% with TBI, 16.9% without) and were the only cohorts with elevations across all OFI categories, including potentially preventable deaths and clinical judgement errors. Care-process adjustment (e.g. emergency interventions, time to radiology, care level) reduced their mortality ORs, suggesting potentially modifiable processes. Isolated severe TBI had the highest mortality (52.8%) but no OFI excess, suggesting outcomes driven by primary injury rather than care-process failures. Penetrating truncal injury showed high, potentially preventable, haemorrhage-related mortality but favourable functional recovery in survivors after care-process adjustment.

**Conclusions:**

Outcome profiles differed across TQIP cohorts and were not captured by mortality alone: isolated severe TBI had the highest mortality but no OFI excess, while the blunt multisystem cohorts had the greatest OFI burden and potentially preventable deaths despite lower mortality. Penetrating truncal injury showed high haemorrhage-related mortality but favourable functional recovery in survivors. Integrating functional outcomes and structured peer review alongside mortality identifies cohorts amenable to quality improvement and reveals cohort-specific processes to target.

**Supplementary Information:**

The online version contains supplementary material available at 10.1186/s13049-026-01674-6.

## Background

Trauma remains a leading cause of death and disability worldwide, causing an estimated 4.9 million deaths in 2023 [1] and the primary cause of death among individuals aged 15 to 49 [2]. To account for the heterogeneity of the trauma population, the American College of Surgeons (ACS) Trauma Quality Improvement Program (TQIP) defines clinically relevant high-risk cohorts for risk-adjusted mortality benchmarking within and across centres [3–5]. In Sweden, cohort-specific benchmarking has confirmed that mortality differences between hospital types are concentrated in specific subgroups rather than reflecting uniform variation in care [6].

However, mortality alone does not distinguish non-preventable from potentially preventable deaths, nor does it capture non-fatal morbidity [7, 8]. Functional outcome measures such as the Glasgow Outcome Scale (GOS) address this gap by capturing morbidity among survivors, though GOS is a crude measure, originally developed for traumatic brain injury with follow-up at 6–12 months [9, 10]. Structured peer review complements mortality and functional outcome by identifying opportunities for improvement (OFIs), preventable errors such as delays in treatment, clinical judgement errors, or missed diagnoses [11–13]. These cohorts are likely to differ not only in expected outcomes but also in the nature of care-process failures and the clinical challenges amenable to intervention.

Characterising these cohort-specific outcome profiles requires combining mortality with measures of morbidity and process errors, yet no previous study has done so across TQIP cohorts. This study aimed to analyse and compare 30-day mortality, cause of death, functional outcomes as measured by the GOS, and OFIs identified through peer review across modified TQIP cohorts.

## Methods

### Study design

We conducted a registry-based cohort study including all trauma patients recorded in both the Karolinska University Hospital trauma registry and the Karolinska trauma care quality database from 1 January 2013 to 28 February 2023. Ethical approval was obtained from the Swedish Ethical Review Authority.

### Setting and participants

The Karolinska University Hospital in Solna is a level 1 equivalent trauma centre and the regional trauma centre for Region Stockholm, managing approximately 1,200 trauma patients annually [14]. The hospital’s trauma registry, part of the Swedish Trauma Registry (SweTrau), includes all patients admitted with trauma team activation, regardless of Injury Severity Score (ISS), those without activation but with ISS > 9, and patients secondarily transferred from other hospitals. The national trauma team activation criteria are summarised in Additional file 11. The registry records data on vital signs, timing, injuries, interventions, demographics, survival status, and functional outcome at discharge from the acute care hospital using the Glasgow Outcome Scale, following the Utstein template [15]. The trauma care quality database captures peer review related data, including audit filters, identified OFIs and their categories, cause of death classifications, and decisions on preventable deaths.

Since 2017, a structured peer review process has been in place: a specialised nurse screens all trauma cases using audit filters [12, 16] and clinical judgement to flag potential OFIs. A second nurse conducts in-depth reviews of flagged cases, which are then assessed in a multidisciplinary peer review conference held bimonthly. All deaths, including those dead on arrival at the hospital (DOA), undergo separate review to determine whether the death was trauma-related, classify cause of death, and evaluate preventability. DOA status is assigned at this review: the guiding definition is death on arrival with no in-hospital intervention undertaken, with the final classification made case by case. Prior to 2017, the review process was informal and clinician-driven. A full description of the review pathway has been published previously [13].

### Eligibility criteria

All patients screened for OFIs in the trauma registry and care quality database during the study period were eligible. Patients under 15 years of age were excluded as they follow separate clinical pathways, and are not included in the peer review process.

### Outcomes

There were three primary outcomes: (1) 30-day all-cause mortality (binary: dead within 30 days of hospital arrival versus alive), (2) unfavourable functional outcome at discharge using the Glasgow Outcome Scale (GOS), dichotomised as unfavourable (GOS 1–3: death, vegetative state, or severe disability) versus favourable (GOS 4–5: moderate disability or good recovery), and (3) the presence of any OFI (binary: yes/no) as determined by consensus during the multidisciplinary peer review conference. Opportunities for improvement were defined as preventable errors in patient care that negatively impact outcomes or deviate from established safe clinical practices, assessed relative to optimal care at the study hospital, the Karolinska University Hospital.

Secondary outcomes included: cause of death (classified through peer review as traumatic brain injury, bleeding, multi-organ failure, or other causes), individual GOS levels (death/vegetative state, severe disability, moderate disability, good recovery), and OFI categories. These were grouped into six categories for analysis: missed diagnosis, delay in treatment, clinical judgement errors, inadequate protocols, inadequate resources, and potentially preventable deaths. Definitions, illustrative examples, and subcategory frequencies for each OFI category are provided in Additional file 1. This categorisation follows previously published work at our centre [12] and comparable external classifications [17].

### Cohort classification

Patients were classified into four mutually exclusive TQIP-based cohorts following previously published definitions [4, 6]: (1) blunt multisystem without TBI, defined as blunt trauma with two or more body regions with Abbreviated Injury Scale (AIS) $\:\ge\:$ 3, without severe TBI; (2) blunt multisystem with TBI, same criteria but including concomitant severe TBI (AIS $\:\ge\:$ 3 head injury); (3) isolated severe TBI, defined as severe TBI with emergency department GCS $\:\le\:$ 8 or prehospital intubation with GCS $\:\le\:$ 8, without multisystem trauma; and (4) penetrating truncal injury, defined as penetrating injuries to central regions (neck, thorax, abdomen) with AIS $\:\ge\:$ 3. Patients not meeting any of these criteria formed the non-TQIP reference cohort.

### Statistical analysis

Baseline characteristics were summarised using medians with interquartile ranges for continuous variables and frequencies with percentages for categorical variables. Associations between cohort membership and each of the three primary outcomes were assessed across four sequential logistic regression models with the non-TQIP cohort as reference level in all models: the unadjusted model estimated crude odds ratios; the patient characteristics model adjusted for age, American Society of Anesthesiologists (ASA) physical status, and sex; the physiological model additionally adjusted for systolic blood pressure and respiratory rate; and the care-process model further adjusted for intubation status, care level, time to emergency procedure, and time to CT (the time-to-procedure and time-to-CT variables, whose reference categories are “no procedure” and “no CT”, also capture whether each was performed). Injury severity (New Injury Severity Score (NISS), AIS) and Glasgow Coma Scale (GCS) were excluded as covariates because they define the cohort classification. The reduction between the physiological and care-process models instead isolates the component of excess risk potentially mediated by modifiable care processes.

Secondary outcomes were assessed using multinomial logistic regression with cohort as the independent variable. Three separate models were fitted: cause of death, modelled as a multinomial outcome where each cause (TBI, bleeding, multi-organ failure, other) was compared against survival as the reference level; GOS level (reference level: good recovery); and OFI category (reference level: no OFI). Covariates from the primary models were not included in the multinomial models due to the rarity of some outcome levels. For the OFI category inadequate protocols, which occurred only in two cohorts, a separate binomial logistic regression was performed.

Because the non-TQIP reference cohort is large and clinically heterogeneous, we ran sensitivity analyses to test whether the cohort odds ratios were sensitive to how this reference group was defined. We described the reference cohort in detail, repeated the analyses using two narrower reference groups (patients with NISS > 15, and patients with a full trauma-team activation), and repeated them once more with the reference removed, comparing the four TQIP cohorts directly against the lowest-mortality cohort. All three are reported in Additional file 10.

Sample size adequacy was evaluated using Riley et al. [18]. The care-process model included 26 parameters with Cox-Snell R² values of 0.238 (mortality), 0.394 (unfavourable GOS), and 0.042 (OFI), yielding minimum required sample sizes of 1,003, 549, and 5,377 respectively (target shrinkage 0.9), all exceeded by our sample of 8,298. Results are reported as odds ratios (OR) with 95% confidence intervals. Analyses were conducted in R (version 4.5.3) [19], with *p* < 0.05 indicating statistical significance.

### Data handling

Missing predictor data were imputed using multiple imputation by chained equations (MICE, 50 iterations) using predictive mean matching (continuous variables and GCS, treated as pseudo-continuous), proportional odds logistic regression (ordinal), logistic regression (binary), and polytomous logistic regression (nominal) [20]. Structural missing values were recoded as explicit factor levels prior to imputation: continuous time-to-event variables were decomposed into a binary indicator (procedure performed yes/no; CT performed yes/no) and an ordered categorical variable for elapsed time (0–30, 31–60, 61–120, > 120 min for emergency procedures; 0–20, 21–30, 31–60, 61–120, > 120 min for CT), with patients who did not undergo the procedure or CT assigned to a “no procedure” or “no CT” level. Outcome variables were included as predictors but not imputed [20, 21]. The maximum fraction of missing information (FMI) was 15.2%, yielding a minimum of m $\:\ge\:$ 16 [22]; we used m = 20 imputed datasets. All models were pooled using Rubin’s rules [23]. Multiple imputation assumes data are missing at random (MAR) conditional on the imputation model—an assumption that cannot be fully verified. To make it more tenable, the imputation model included the outcomes and a broad predictor set spanning patient, physiological, and care-process variables. Convergence was assessed visually via trace plots and quantitatively using the potential scale reduction factor (R-hat) [24] (Additional file 8). All R-hat values were below 1.05 at the final iteration.

## Results

### Participants

**Fig. 1:**
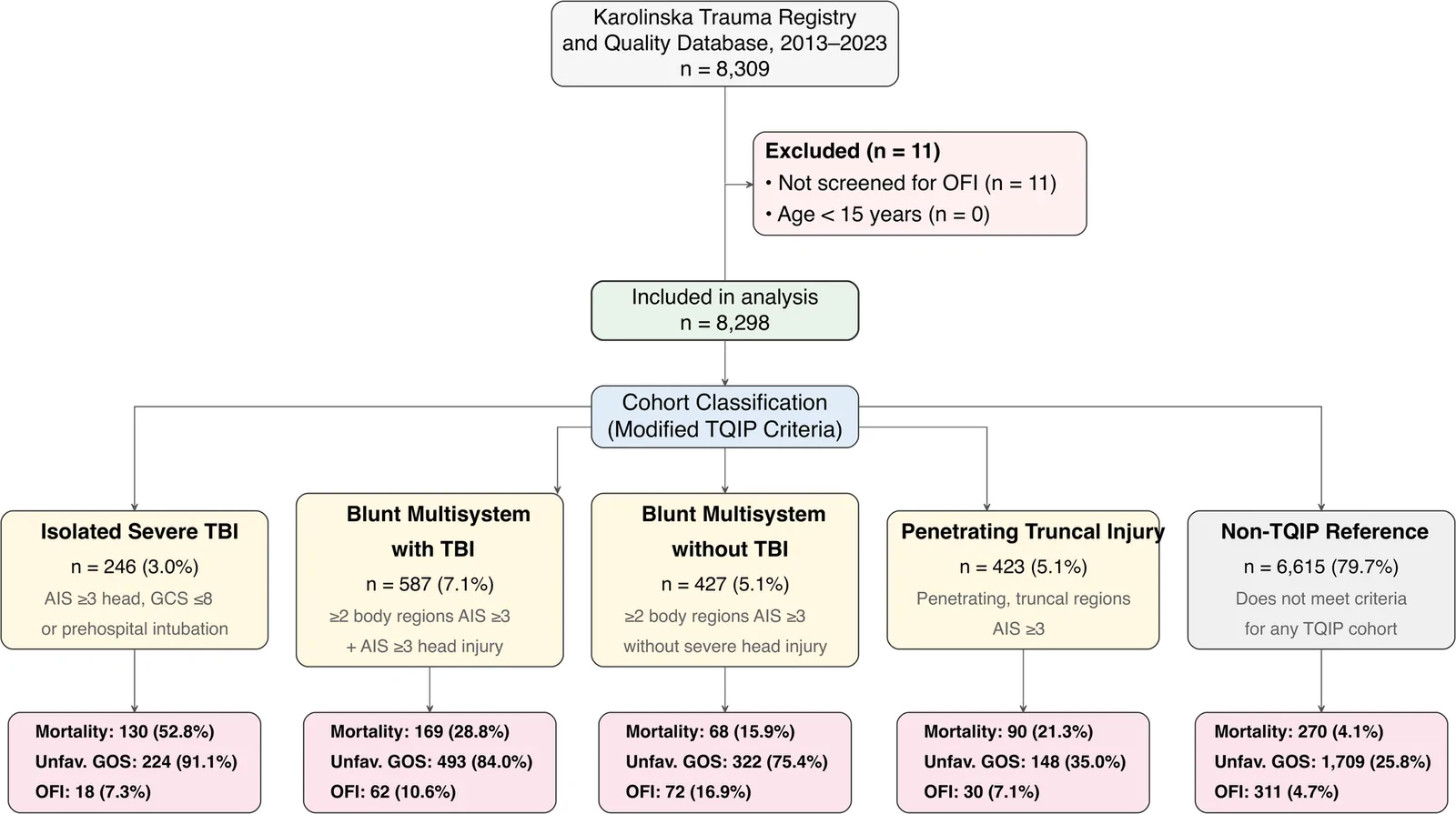
Study flowchart showing patient inclusion, cohort classification, and outcomes. Cohort percentages = % of total included patients. Outcome percentages = % within each cohort. AIS = Abbreviated Injury Scale; GCS = Glasgow Coma Scale; GOS = Glasgow Outcome Scale; OFI = Opportunity for Improvement; TBI = Traumatic Brain Injury; TQIP = Trauma Quality Improvement Program

This study included 8,298 patients, of whom 5,748 (69.3%) were male. Patients were categorised into the five cohorts: isolated severe TBI (*n* = 246, 3%), blunt multisystem with TBI (*n* = 587, 7.1%), blunt multisystem without TBI (*n* = 427, 5.1%), penetrating truncal injury (*n* = 423, 5.1%), and the non-TQIP reference cohort (*n* = 6,615, 79.7%). The median age was 43 (IQR 27, 61) years, with the youngest cohort being patients with penetrating truncal injury (28 (IQR 21, 41) years) and the oldest being patients with isolated severe TBI (59 (IQR 38, 74) years). The median NISS was 11 (IQR 3, 22). The study flowchart is presented in Fig. 1. Full baseline characteristics and outcome distributions, together with the proportion of missing data per variable before imputation, are shown in Table 1.

**Table 1:** Baseline characteristics and outcomes by trauma cohort

	TQIP Cohort					
	Overall *N* = 8,298^1^	Isolated TBI *N* = 246^1^	BM with TBI *N* = 587^1^	BM without TBI *N* = 427^1^	Penetrating truncal injury *N* = 423^1^	Non TQIP-cohort *N* = 6,615^1^
**Age**,** years**	43 (27, 61)	59 (38, 74)	49 (30, 66)	49 (32, 64)	28 (21, 41)	42 (27, 61)
**Male sex**	5,748 (69%)	183 (74%)	409 (70%)	305 (71%)	382 (90%)	4,469 (68%)
**ASA ≥ 3**	1,539 (19%)	81 (33%)	138 (24%)	88 (21%)	29 (7.0%)	1,203 (18%)
Missing	27	4	5	4	6	8
**NISS**	11 (3, 22)	38 (27, 50)	34 (27, 50)	34 (27, 41)	22 (14, 35)	8 (3, 17)
Missing	11	0	0	0	0	11
**Intubated in ED**	725 (8.7%)	130 (53%)	133 (23%)	67 (16%)	93 (22%)	302 (4.6%)
Missing	2	0	1	0	0	1
**Respiratory rate**	18 (16, 20)	18 (14, 20)	18 (16, 20)	18 (16, 22)	18 (17, 22)	18 (16, 20)
Missing	868	105	244	86	107	326
**SBP**,** mmHg**	135 (120, 150)	143 (120, 165)	128 (100, 146)	125 (104, 142)	120 (83, 141)	136 (122, 150)
Missing	13	0	1	4	1	7
**GCS**	15 (14, 15)	5 (3, 6)	14 (10, 15)	15 (14, 15)	15 (15, 15)	15 (15, 15)
Missing	865	97	241	88	105	334
**Admission ward**						
ED	1,499 (18%)	9 (3.7%)	40 (6.8%)	20 (4.7%)	33 (7.8%)	1,397 (21%)
General ward	3,076 (37%)	18 (7.3%)	78 (13%)	50 (12%)	65 (15%)	2,865 (43%)
Operating theatre	1,590 (19%)	3 (1.2%)	81 (14%)	137 (32%)	147 (35%)	1,222 (18%)
Specialised ward	393 (4.7%)	2 (0.8%)	20 (3.4%)	25 (5.9%)	23 (5.4%)	323 (4.9%)
ICU	1,740 (21%)	214 (87%)	368 (63%)	195 (46%)	155 (37%)	808 (12%)
**Emergency intervention**						
No intervention	5,970 (72%)	135 (55%)	283 (48%)	174 (41%)	69 (16%)	5,309 (80%)
Thoracotomy	105 (1.3%)	3 (1.2%)	14 (2.4%)	16 (3.7%)	59 (14%)	13 (0.2%)
Laparotomy	245 (3.0%)	0 (0%)	23 (3.9%)	33 (7.7%)	104 (25%)	85 (1.3%)
Pelvic packing	5 (< 0.1%)	0 (0%)	1 (0.2%)	2 (0.5%)	0 (0%)	2 (< 0.1%)
Revascularisation	49 (0.6%)	0 (0%)	2 (0.3%)	5 (1.2%)	3 (0.7%)	39 (0.6%)
Radiological intervention	101 (1.2%)	0 (0%)	17 (2.9%)	31 (7.3%)	2 (0.5%)	51 (0.8%)
Craniotomy	283 (3.4%)	64 (26%)	50 (8.5%)	0 (0%)	1 (0.2%)	168 (2.5%)
ICP monitoring only	102 (1.2%)	40 (16%)	35 (6.0%)	0 (0%)	0 (0%)	27 (0.4%)
Other action§	1,438 (17%)	4 (1.6%)	162 (28%)	166 (39%)	185 (44%)	921 (14%)
**Time to procedure**,** min**	106 (54, 257)	114 (80, 163)	98 (19, 160)	100 (16, 245)	30 (10, 80)	134 (79, 419)
Not performed	5,970	135	283	174	69	5,309
**Time to CT**,** min**	33 (21, 66)	27 (21, 37)	32 (23, 47)	35 (24, 58)	32 (22, 54)	34 (21, 71)
Not performed	998	9	76	83	110	720
30-day mortality*	727 (8.8%)	130 (53%)	169 (29%)	68 (16%)	90 (21%)	270 (4.1%)
30-day mortality excl. DOA*	564 (6.8%)	122 (50%)	128 (22%)	46 (11%)	39 (9.2%)	229 (3.5%)
**Cause of death**,** n (% cohort**,** % deaths)†**						
Bleeding	131 (1.6%, 18.6%)	0	21 (3.6%, 12.4%)	28 (6.6%, 41.2%)	71 (16.8%, 78.9%)	11 (0.2%, 4.4%)
Multi-organ failure	36 (0.4%, 5.1%)	0	3 (0.5%, 1.8%)	12 (2.8%, 17.6%)	1 (0.2%, 1.1%)	20 (0.3%, 8.0%)
Other	183 (2.2%, 26.0%)	7 (2.8%, 5.5%)	28 (4.8%, 16.6%)	22 (5.2%, 32.4%)	5 (1.2%, 5.6%)	121 (1.8%, 48.4%)
TBI	329 (4.0%, 46.7%)	119 (48.4%, 93.7%)	109 (18.6%, 64.5%)	6 (1.4%, 8.8%)	13 (3.1%, 14.4%)	82 (1.2%, 32.8%)
Unknown	25 (0.3%, 3.6%)	1 (0.4%, 0.8%)	8 (1.4%, 4.7%)	0	0	16 (0.2%, 6.4%)
GOS at discharge*						
Good recovery	2,629 (32%)	3 (1.2%)	1 (0.2%)	4 (0.9%)	59 (14%)	2,562 (39%)
Moderate disability	2,773 (33%)	19 (7.7%)	93 (16%)	101 (24%)	216 (51%)	2,344 (35%)
Severe disability	2,225 (27%)	91 (37%)	325 (55%)	253 (59%)	57 (13%)	1,499 (23%)
Dead/Vegetative	671 (8.1%)	133 (54%)	168 (29%)	69 (16%)	91 (22%)	210 (3.2%)
**OFI identified**	493 (5.9%)	18 (7.3%)	62 (11%)	72 (17%)	30 (7.1%)	311 (4.7%)
**OFI type**,** n (% cohort**,** % OFIs)‡**						
Clinical judgement error	174 (2.1%, 35.3%)	7 (2.8%, 38.9%)	25 (4.3%, 40.3%)	30 (7.0%, 41.7%)	12 (2.8%, 40.0%)	100 (1.5%, 32.2%)
Delay in treatment	72 (0.9%, 14.6%)	5 (2.0%, 27.8%)	7 (1.2%, 11.3%)	9 (2.1%, 12.5%)	5 (1.2%, 16.7%)	46 (0.7%, 14.8%)
Inadequate protocols	24 (0.3%, 4.9%)	0	2 (0.3%, 3.2%)	7 (1.6%, 9.7%)	0	15 (0.2%, 4.8%)
Inadequate resources	111 (1.3%, 22.5%)	2 (0.8%, 11.1%)	13 (2.2%, 21.0%)	14 (3.3%, 19.4%)	6 (1.4%, 20.0%)	76 (1.1%, 24.4%)
Missed diagnosis	70 (0.8%, 14.2%)	2 (0.8%, 11.1%)	7 (1.2%, 11.3%)	7 (1.6%, 9.7%)	1 (0.2%, 3.3%)	53 (0.8%, 17.0%)
Potentially preventable deaths	42 (0.5%, 8.5%)	2 (0.8%, 11.1%)	8 (1.4%, 12.9%)	5 (1.2%, 6.9%)	6 (1.4%, 20.0%)	21 (0.3%, 6.8%)

^1^Median (Q1, Q3) for continuous; n (%) for categorical

§ Other action includes: chest drain, external fracture fixation, major fracture surgery, wound revision in OR, other procedure

† Cause of death: n (% of cohort, % of deaths in cohort)

‡ OFI type: n (% of cohort, % of OFIs in cohort)

* GOS is assessed at discharge from the primary admitting hospital; 30-day mortality is ascertained independently. The two measures are not directly comparable, as patients discharged alive may die within 30 days

“Missing” rows show the number and percentage of patients with missing data for each variable before multiple imputation. Cause of death and OFI type are not applicable for survivors and non-OFI patients, respectively, and are not shown as missing. Time to procedure and time to CT are medians among patients who underwent the procedure or CT; “Not performed” counts those who did not (entered into the models as an explicit category rather than as missing data, see Methods)

### Mortality and cause of death

**Fig. 2:**
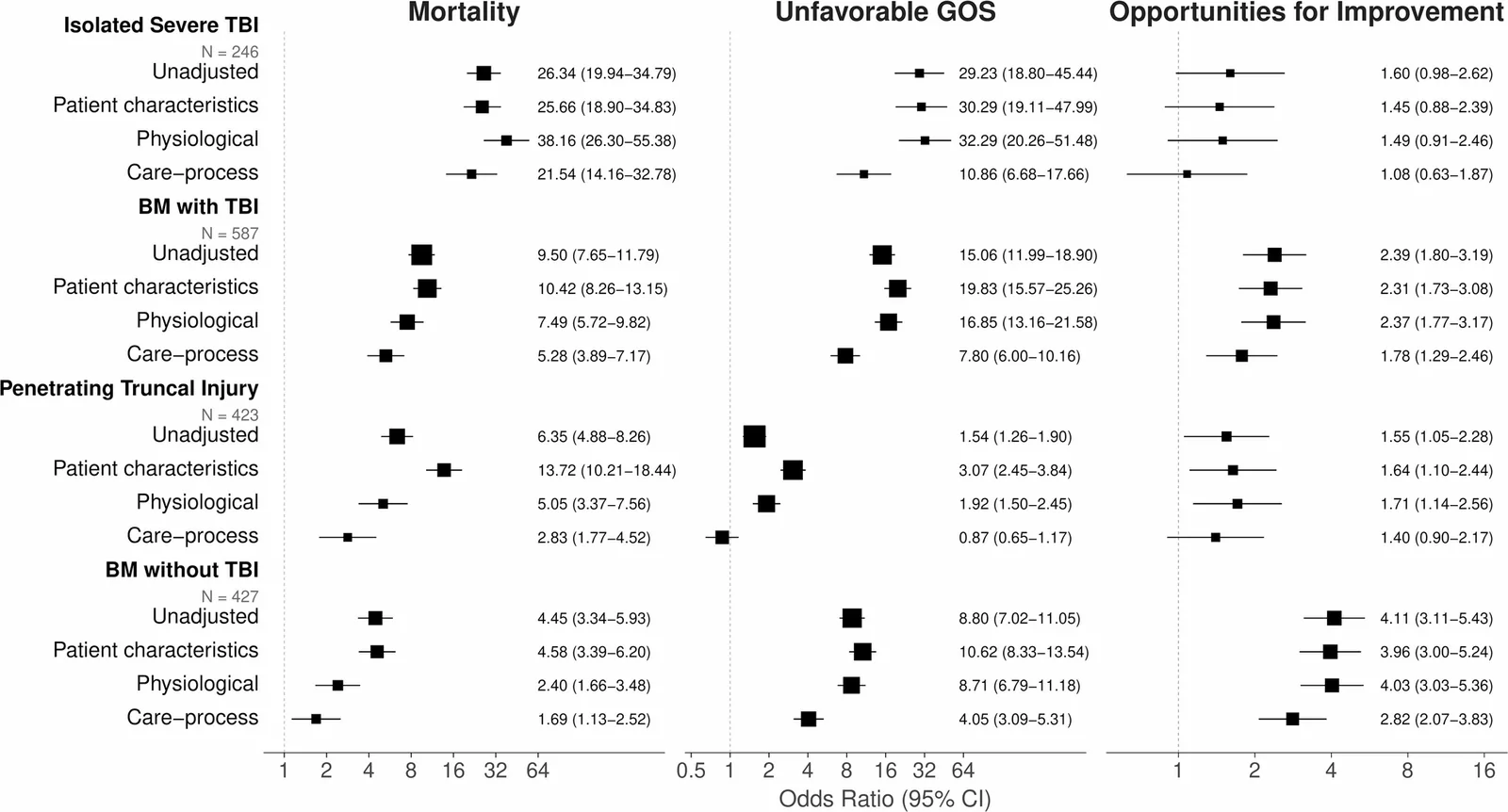
Forest plot of odds ratios from cumulative sequential logistic regression models for 30-day mortality, unfavourable GOS, and opportunities for improvement. Each model retains all covariates from the preceding step. Reference: Non TQIP-cohort. Each model includes all covariates from previous steps. Unadjusted: cohort only. Patient characteristics: + age, ASA, sex. Physiological: + SBP, respiratory rate. Care-process: + intubation, care level, time to procedure, time to CT (the time variables also encode whether the procedure/CT was performed)

Overall, 727 patients (8.8%) died within 30 days. Mortality ranged from 15.9% in blunt multisystem without TBI to 52.8% in isolated severe TBI, compared with 4.1% in the non-TQIP reference cohort (Table 1). Excluding DOA, the largest absolute mortality reduction was in penetrating truncal injury (21.3% to 9.2%) and the smallest in isolated severe TBI (52.8% to 49.6%). Unadjusted odds of mortality were significantly increased in all TQIP cohorts and remained elevated after sequential adjustment for patient characteristics, physiological status, and care processes (Fig. 2, Additional files 2–5). The care-process adjustment step reduced ORs across all cohorts, most notably in blunt multisystem without TBI (Fig. 2).

**Fig. 3:**
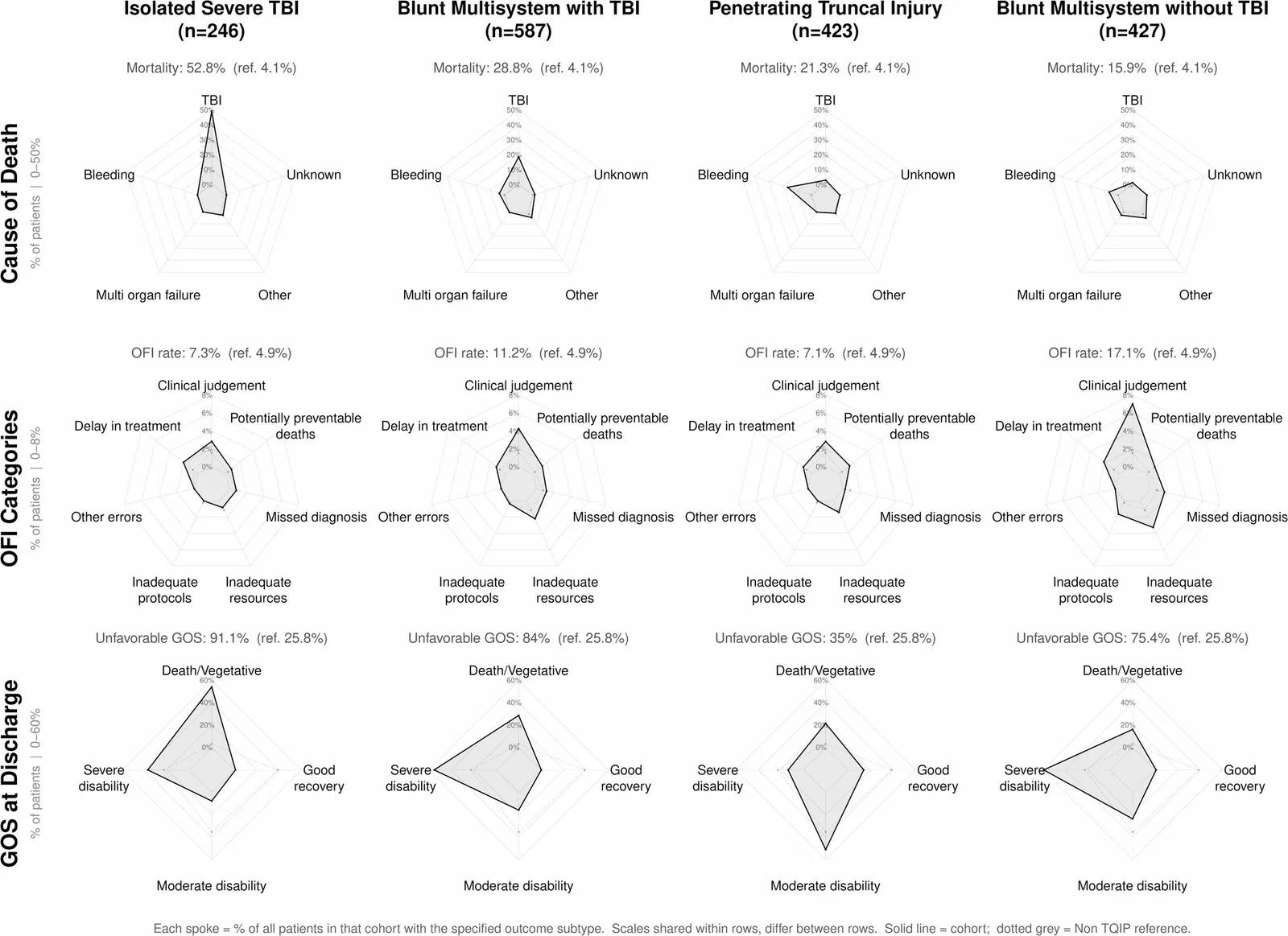
Radar charts of outcome category distributions by TQIP cohort. Percentage of patients in each cohort with specific outcome categories for cause of death, OFI categories, and GOS levels. Solid lines represent TQIP cohorts; dotted grey lines represent the non-TQIP reference

Among deaths with a classified cause, TBI was the most common overall, followed by bleeding and multi-organ failure. Cause-of-death patterns differed across cohorts (Fig. 3, Additional file 6). Deaths in the TBI-associated cohorts were predominantly TBI-related, particularly in isolated severe TBI (94%), whereas blunt multisystem without TBI showed a heterogeneous distribution across bleeding, multi-organ failure, and other causes. In penetrating truncal injury, bleeding was the dominant cause.

### Functional outcomes

Unfavourable GOS (< 4) at discharge was recorded in 2,896 patients overall. Among the TQIP cohorts, rates ranged from 35% in penetrating truncal injury to 91.1% in isolated severe TBI, compared with 25.8% in the reference cohort (Table 1). Compared to the non-TQIP cohort, all TQIP cohorts had increased unadjusted odds of unfavourable GOS, with the highest OR in isolated severe TBI (29.23 (18.80–45.44)). These associations persisted across adjustment levels, though ORs were substantially reduced after adjusting for care processes (Fig. 2, Additional files 3, 5). The OR for penetrating truncal injury crossed below 1.0 after adjusting for care processes (OR 0.87 (0.65–1.17)), while isolated severe TBI remained markedly elevated (OR 10.86 (6.68–17.66)). Among survivors, penetrating truncal injury had the highest proportion of good recovery (13.9%), whereas severe disability predominated in the blunt multisystem cohorts (Additional file 7).

### Opportunities for improvement

Opportunities for improvement were identified in 493 patients (5.9%), with rates highest in the blunt multisystem cohorts (Table 1). Blunt multisystem without TBI had the highest unadjusted OFI elevation (OR 4.11 (3.11–5.43)), persisting across all adjustment levels, and was the only cohort with significantly elevated odds across all OFI categories, including inadequate protocols, which was not significantly elevated in any other cohort (Fig. 2; Table 2). Isolated severe TBI showed no significant overall OFI excess despite the highest mortality, though the category analysis identified elevated odds of delays in treatment. In penetrating truncal injury and blunt multisystem with TBI, clinical judgement errors and potentially preventable deaths were the predominant categories, with blunt multisystem with TBI showing the highest odds of potentially preventable deaths across all cohorts (Table 2). Each TQIP cohort thus displayed a distinct outcome profile across mortality, functional outcomes, and OFI categories (Fig. 3).

These cohort differences were consistent in the sensitivity analyses: the higher-mortality cohorts remained elevated when the reference was restricted to the most severely injured patients (NISS > 15) or to those with a full trauma-team activation (Trauma-1), and the mortality–OFI dissociation was reproduced when the reference cohort was removed and the four TQIP cohorts were compared directly. The full sensitivity analyses, including a characterisation of the reference cohort, are reported in Additional file 10 (Tables S10a–c).

**Table 2:** OFI categories by TQIP cohort

Cohort	OFI category	OFI Frequency	OR	95% CI	*p*-value
Blunt multisystem without TBI (*n* = 427)	No OFI	354 / 7,788 (4.5%)	—		
	Clinical judgement error	30 / 174 (17.2%)	5.33	3.50, 8.13	**< 0.001**
	Delay in treatment	9 / 72 (12.5%)	3.48	1.69, 7.16	**< 0.001**
	Inadequate protocols	7 / 24 (29.2%)	7.34	2.79, 17.5	**< 0.001**
	Inadequate resources	14 / 111 (12.6%)	3.27	1.83, 5.85	**< 0.001**
	Missed diagnosis	7 / 70 (10.0%)	2.35	1.06, 5.20	**0.035**
	Potentially preventable deaths	5 / 42 (11.9%)	4.23	1.59, 11.29	**0.004**
Blunt multisystem with TBI (*n* = 587)	No OFI	521 / 7,788 (6.7%)	—		
	Clinical judgement error	25 / 174 (14.4%)	3.02	1.93, 4.72	**< 0.001**
	Delay in treatment	7 / 72 (9.7%)	1.84	0.83, 4.09	0.136
	Inadequate resources	13 / 111 (11.7%)	2.07	1.14, 3.75	**0.017**
	Missed diagnosis	7 / 70 (10.0%)	1.60	0.72, 3.53	0.249
	Potentially preventable deaths	8 / 42 (19.0%)	4.60	2.03, 10.44	**< 0.001**
Isolated severe TBI (*n* = 246)	No OFI	228 / 7,788 (2.9%)	—		
	Clinical judgement error	7 / 174 (4.0%)	1.93	0.89, 4.20	0.097
	Delay in treatment	5 / 72 (6.9%)	3.00	1.18, 7.62	**0.021**
	Inadequate resources	2 / 111 (1.8%)	0.73	0.18, 2.97	0.656
	Missed diagnosis	2 / 70 (2.9%)	1.04	0.25, 4.30	0.955
	Potentially preventable deaths	2 / 42 (4.8%)	2.63	0.61, 11.28	0.193
Penetrating truncal injury (*n* = 423)	No OFI	393 / 7,788 (5.0%)	—		
	Clinical judgement error	12 / 174 (6.9%)	1.92	1.05, 3.53	**0.035**
	Delay in treatment	5 / 72 (6.9%)	1.74	0.69, 4.40	0.242
	Inadequate resources	6 / 111 (5.4%)	1.26	0.55, 2.92	0.583
	Missed diagnosis	1 / 70 (1.4%)	0.30	0.04, 2.19	0.236
	Potentially preventable deaths	6 / 42 (14.3%)	4.57	1.84, 11.40	**0.001**

Odds ratios relative to the non-TQIP reference cohort. Bold indicates *p* < 0.05. OFI Frequency: n / N (%), where N is the total count of that OFI category across all cohorts. Multinomial logistic regression for all categories except Inadequate protocols (binomial; only present in two cohorts). Other errors excluded

## Discussion

This study examined 30-day mortality, functional outcome (GOS), and OFIs across modified TQIP cohorts. All TQIP cohorts carried substantially elevated risks of both mortality and unfavourable functional outcomes that persisted after sequential adjustment. Care-process adjustment reduced ORs across all cohorts, but this statistical signal converged with elevated OFI rates only in the blunt multisystem cohorts, identifying them as the populations where quality improvement efforts have the greatest potential.

Blunt multisystem without TBI had the highest OFI rate, the only significant elevations across all OFI categories, and a reduced mortality OR after adjusting for care processes (OR 2.4 to 1.69) consistent with partial mediation by modifiable care processes, though this reflects association rather than proven causation. Despite a median NISS of 34, these patients presented with apparent physiological stability: a median GCS of 15, median SBP of 125 mmHg, median respiratory rate of 18 breaths/min, and the lowest ICU admission rate among the TQIP cohorts (46%). Yet their multisystem injuries require complex interventions across multiple organ systems — 59% underwent emergency procedures, spanning the widest range of intervention types across cohorts (Table 1) — creating more decision points where errors in clinical judgement, protocols, and resource allocation can occur. This complexity, combined with the apparent physiological stability, may compound the risk by leading to underestimation of injury severity and insufficient escalation of care, ultimately contributing to potentially preventable deaths [25, 26]. The cause-of-death distribution in this cohort was notably heterogeneous, with multi-organ failure featuring prominently alongside bleeding, consistent with a pattern in which initially stable patients deteriorate over time without adequate monitoring and management. That multi-organ failure was a substantial cause of death even though these patients less often received intensive care, combined with elevated OFIs in care level and inadequate resources, suggests that earlier escalation to higher-level care, with appropriate management, could mitigate some preventable deterioration—though some of these late deaths follow mechanisms that remain incompletely understood and difficult to prevent even with optimal contemporary care [27].

Beyond mortality, the blunt multisystem cohorts carried a disproportionate burden of unfavourable functional outcomes, signalling morbidity and rehabilitation needs that mortality statistics do not capture. Elevated odds of missed injuries may contribute to both delayed deterioration and the high proportion of patients discharged with severe disability. Longitudinal data from the same centre show that blunt multisystem without TBI remains the only cohort with persistently elevated OFI risk despite an overall decrease in OFI incidence over time [28]. Blunt multisystem with TBI showed a similar but less pronounced pattern, with OFIs predominantly driven by clinical judgement errors and potentially preventable deaths, consistent with the complexity of managing concurrent neurosurgical and general surgical priorities [29].

In contrast, isolated severe TBI showed no significant overall OFI excess despite the highest mortality (52.8%) and unfavourable GOS rate. Adjusting for care processes reduced ORs but, in the absence of elevated OFI rates, this likely reflects necessary care responses (ICU admission and intubation) rather than modifiable quality gaps. Deaths were predominantly TBI-related (94%), and this cohort nonetheless had the lowest proportion of DOA among the TQIP cohorts (8 [3.3%]), reflecting injuries that are not acutely lethal but progress over hours to days — consistent with the paradoxical increase in mortality OR after adjusting for physiological status, which confirms that preserved haemodynamics on presentation do not exclude severe brain injury. Nevertheless, the category analysis identified significantly elevated odds of delays in treatment. Although CT times were the fastest across cohorts, time to definitive neurosurgical intervention was prolonged, suggesting that reducing this interval is the principal modifiable target in a cohort where the primary brain injury otherwise largely determines outcomes. The absence of elevated odds of potentially preventable deaths aligns with the preventable death literature, in which severe TBI is consistently classified as the predominant cause of non-preventable trauma mortality [17, 30], and with prognostic studies identifying initial neurological status as the dominant outcome determinant [31, 32].

Penetrating truncal injury presented a distinctive bimodal pattern of high mortality alongside favourable functional recovery in survivors, reflecting injuries where haemorrhage is either rapidly lethal, as evidenced by the highest proportion of DOA (51 [12.1%]), or surgically controllable [33]. After adjusting for care processes, the OR for unfavourable GOS fell below 1.0, placing functional recovery in survivors on par with—or better than—the lower-severity non-TQIP reference cohort, and pointing to fast, definitive surgical haemostasis, with the shortest time to intervention across cohorts, as the likely driver. The mortality OR increased after adjusting for patient characteristics (from 6.35 to 13.72), indicating that the young, low-comorbidity profile of these patients masks the true lethality of the injuries; once this demographic advantage is accounted for, the excess mortality is even greater. Although the overall OFI elevation was reduced after full adjustment, the OFI category analysis revealed significantly elevated odds for clinical judgement errors and potentially preventable deaths. Bleeding was the dominant cause of death, and haemorrhage is consistently identified as the leading cause of potentially preventable trauma mortality [17, 34, 35]. The co-occurrence of haemorrhage-dominated deaths with elevated odds of clinical judgement errors and potentially preventable deaths suggests that care failures in this cohort may carry disproportionate consequences [36].

The inverse relationship between mortality and OFI risk challenges the use of mortality as a sole quality indicator [37]. The TQIP framework uses risk-adjusted mortality benchmarking across centres to identify underperformance [4], but this approach can detect that a cohort underperforms without characterising why. As illustrated by the blunt multisystem without TBI cohort, care-process failures that manifest primarily as morbidity rather than death may go unrecognised, consistent with evidence from our centre that opportunities for improvement concentrate in physiologically stable patients who survive [38] and that mortality-based case selection fails to capture them [13]. Gruen et al. [34] identified distinct error patterns contributing to trauma mortality concentrated in specific phases of care; our findings extend this by demonstrating that the distribution of OFIs across cohorts is not proportional to mortality. Preventable death reviews remain valuable [7], but mortality alone does not capture the full spectrum of care-process failures. Integrating functional outcomes and process indicators alongside mortality is necessary for comprehensive quality assessment.

### Strengths and limitations

This study benefits from a large cohort spanning 10 years with structured peer review and simultaneous assessment of mortality, functional outcomes, and process errors across standardised trauma cohorts. The sequential modelling framework introduces care-process variables in the final step, which enabled us to estimate the excess risk potentially attributable to modifiable processes. Additionally, the peer review process may itself contribute to quality improvement through a Hawthorne effect: the awareness that all cases are subject to systematic screening and multidisciplinary peer review can drive behavioural change among all healthcare personnel, independent of the specific errors identified.

Several limitations merit consideration. The OFI identification relies on audit filters and nurse-led case reviews with limited individual predictive ability [16, 39], and no combination of filters captures all care-process errors. Observed OFI rates may therefore partly reflect detection bias, where cohorts with previously identified high-impact OFIs receive sustained scrutiny while others may be under-scrutinised; a low observed OFI rate should not be interpreted as confirmation that care-process errors are absent. Because the peer review process was formalised only in 2017, its greater sensitivity may have increased OFI detection over time, contributing to temporal variation independent of any true change in care.

Additionally, some OFI categories had limited case numbers, particularly in the smaller cohorts, and the resulting effect estimates should be interpreted as signals identifying areas for quality improvement attention rather than precise measures of magnitude. The GOS was assessed at discharge from the primary admitting hospital; discharge includes transfer to another facility, home, or death. Discharge GOS therefore reflects functional status at a variable and often early timepoint rather than at the standardised 6–12 month follow-up for which the scale was designed, and may over- or underestimate long-term disability. Finally, the models estimate associations rather than causal effects; the odds-ratio reductions after care-process adjustment reflect statistically attributable risk, and residual confounding cannot be excluded.

When comparing DOA-excluded mortality rates with national Swedish data (NISS > 15) [6], isolated severe TBI (49.6% versus 50%) and blunt multisystem without TBI (10.8% versus 9%) were comparable, supporting the external validity of findings in these cohorts. Blunt multisystem with TBI mortality was higher (21.8% versus 12%), likely reflecting the injury severity at a level 1 referral centre, and penetrating trauma mortality was lower (9.2% versus 20%), consistent with high-volume surgical haemostasis capacity. The comparatively high proportion of penetrating truncal injury and the small isolated severe TBI cohort—atypical of many European cohorts—reflect the urban catchment of a metropolitan Scandinavian level 1 centre and the strict, mutually exclusive cohort definitions (isolated severe TBI excludes TBI patients who also meet multisystem criteria). These case-mix differences should be considered when generalising to other settings.

## Conclusions

Outcome profiles differed markedly across TQIP cohorts and were not captured by mortality alone: isolated severe TBI had the highest mortality yet one of the lowest OFI rates, while the blunt multisystem cohorts, particularly without TBI, had the greatest OFI burden and potentially preventable deaths despite lower mortality. Penetrating truncal injury showed high haemorrhage-related mortality but favourable functional recovery in survivors. Risk-adjusted mortality benchmarking alone cannot characterise these differences. Integrating functional outcomes and structured peer review alongside mortality not only identifies which cohorts are amenable to quality improvement but also reveals potential cohort-specific processes to target.

## Supplementary Information

Below is the link to the electronic supplementary material.

Supplementary Material 1

## Data Availability

The datasets analysed during the current study are not publicly available due to containing sensitive patient information protected under Swedish law. Data access requests can be directed to the corresponding body at Karolinska University Hospital and are subject to approval by the Swedish Ethical Review Authority.

## Abbreviations

ACS: American College of Surgeons
ASA: American Society of Anesthesiologists
CI: Confidence interval
CT: Computed tomography
GCS: Glasgow Coma Scale
GOS: Glasgow Outcome Scale
ICU: Intensive care unit
ISS: Injury Severity Score
NISS: New Injury Severity Score
OFI: Opportunity for improvement
OR: Odds ratio
SweTrau: Swedish Trauma Registry
TBI: Traumatic brain injury
TQIP: Trauma Quality Improvement Program
