# Supplementary material for: Clinical outcomes and opportunities for improvement across modified trauma quality improvement cohorts: a registry-based cohort study
Source: Scand J Trauma Resusc Emerg Med. 2026 Aug 17;34:140. doi: 10.1186/s13049-026-01674-6 (PMC13495265; doi:10.1186/s13049-026-01674-6)
Supplement: Supplementary file 1 [file 13049_2026_1674_MOESM1_ESM.docx]

Supplementary material

**Supplementary material for:**

**Clinical outcomes and opportunities for improvement across modified trauma quality improvement cohorts: a registry-based cohort study**

Jonatan Attergrim^1,2^, Lovisa Strömmer^1,3^, Olof Brattström^4^, Martin Jacobsson^5^, Josef Al-Khalili^1^, Elin Sun Cao^1^, Kelvin Szolnoky^6^, Johanna Berg^1,7^, Martin Gerdin Wärnberg^1,2^

^1^ Department of Global Public Health, Karolinska Institutet, Stockholm, Sweden
^2^ Perioperative Medicine and Intensive Care, Karolinska University Hospital, Solna, Stockholm, Sweden
^3^ Department of Clinical Science, Intervention and Technology, Division of Surgery, Karolinska Institutet, Karolinska University Hospital Huddinge, Stockholm, Sweden
^4^ Department of Anesthesiology, Mora Hospital, Mora, Sweden
^5^ Department of Biomedical Engineering and Health Systems, KTH Royal Institute of Technology, Huddinge, Sweden
^6^ Department of Medical Epidemiology and Biostatistics, Karolinska Institutet, Stockholm, Sweden
^7^ Department of Internal and Emergency Medicine, Skånes universitetssjukhus Malmö, Malmö, Sweden

**Corresponding author:** Jonatan Attergrim, Department of Global Public Health, Karolinska Institutet, 171 77 Stockholm, Sweden. Email: jonatan.attergrim@ki.se

This document contains the additional files referenced in the main manuscript.

**Contents**

Additional file 1: OFI categories with definitions and subcategory frequencies

Additional file 2: Full logistic regression results: 30-day mortality

Additional file 3: Full logistic regression results: Unfavourable GOS

Additional file 4: Full logistic regression results: Opportunities for improvement

Additional file 5: Unadjusted and fully adjusted odds ratios by cohort

Additional file 6: Cause of death by TQIP cohort (multinomial logistic regression)

Additional file 7: Individual GOS levels by TQIP cohort (multinomial logistic regression)

Additional file 8: MICE convergence diagnostics

Additional file 9: OR trajectories across sequential models

Additional file 10: Reference cohort composition and sensitivity analyses

Additional file 11: National trauma team activation criteria

# Additional files

## Additional file 1: OFI categories with definitions and subcategory frequencies

| **Category (n)** | **Opportunities for Improvement, N = 493** |
| --- | --- |
| **Potentially preventable deaths (42)** Deaths judged by peer review as preventable or potentially avoidable given optimal care at the study hospital. | **• Potentially preventable death:** 38 *e.g., death where alternative management may have altered the outcome* **• Preventable death:** 4 *e.g., death from surgically accessible haemorrhage where timely intervention was feasible* |
| **Missed diagnosis (70)** Failure to identify an injury or condition despite sufficient information and resources. | **• Missed injury:** 69 *e.g., injury not identified on imaging or failure to perform indicated radiology* **• Tertiary survey:** 1 *e.g., injury detected only after a delayed or omitted tertiary survey* |
| **Delay in treatment (72)** Inappropriate delay from assessment to treatment or imaging, not attributable to resource constraints. | **• Delay to surgery:** 47 *e.g., prolonged time to intervention not attributable to incorrect management or lacking resources* **• Delay to CT:** 25 *e.g., delayed CT in a patient with reduced consciousness and later confirmed intracranial injury* |
| **Clinical judgement error (174)** Incorrect management strategy despite sufficient information and resources. | **• Triage in the ED:** 63 *e.g., highest trauma level deprioritised despite clinical signs of instability* **• Patient management:** 48 *e.g., no epidural analgesia for multiple rib fractures, or CT performed before surgery when immediate intervention was indicated* **• Level of care:** 47 *e.g., intermediate care when ICU indicated, including inter-hospital transfers* **• Communication:** 16 *e.g., failed handover between teams* |
| **Inadequate protocols (24)** Absent or insufficient protocols or guidelines. | **• Trauma criteria/guidelines:** 22 *e.g., protocol undertriage of patient* **• Inadequate routine:** 2 *e.g., absent or unclear local clinical guidelines for managing a specific injury pattern* |
| **Inadequate resources (111)** Lack of personnel, material, or infrastructure. | **• Resources:** 73 *e.g., no operating room available* **• Logistics/technical:** 36 *e.g., IT system or equipment failure* **• Specialist availability:** 2 *e.g., required specialist unavailable* |

## Additional file 2: Full logistic regression results: 30-day mortality

| Term | Unadjusted OR (95% CI) | Unadj. p | Patient char. OR (95% CI) | Patient char. p | Physiological OR (95% CI) | Physiological p | Care-process OR (95% CI) | Care-process p |
| --- | --- | --- | --- | --- | --- | --- | --- | --- |
| Cohort: blunt multisystem with TBI | 9.50 (7.65–11.79) | <0.001 | 10.42 (8.26–13.15) | <0.001 | 7.49 (5.72–9.82) | <0.001 | 5.28 (3.89–7.17) | <0.001 |
| Cohort: blunt multisystem without TBI | 4.45 (3.34–5.93) | <0.001 | 4.58 (3.39–6.20) | <0.001 | 2.40 (1.66–3.48) | <0.001 | 1.69 (1.13–2.52) | 0.011 |
| Cohort: Isolated severe TBI | 26.34 (19.94–34.79) | <0.001 | 25.66 (18.90–34.83) | <0.001 | 38.16 (26.30–55.38) | <0.001 | 21.54 (14.16–32.78) | <0.001 |
| Cohort: severe penetrating | 6.35 (4.88–8.26) | <0.001 | 13.72 (10.21–18.44) | <0.001 | 5.05 (3.37–7.56) | <0.001 | 2.83 (1.77–4.52) | <0.001 |
| Age (years) |  |  | 1.04 (1.03–1.04) | <0.001 | 1.05 (1.04–1.06) | <0.001 | 1.06 (1.05–1.06) | <0.001 |
| ASA: 2 |  |  | 0.61 (0.48–0.79) | <0.001 | 0.70 (0.52–0.94) | 0.019 | 0.68 (0.50–0.92) | 0.014 |
| ASA: 3 |  |  | 1.42 (1.10–1.83) | 0.007 | 1.67 (1.25–2.24) | <0.001 | 1.63 (1.20–2.21) | 0.002 |
| ASA: 4 |  |  | 4.96 (2.86–8.60) | <0.001 | 5.70 (3.14–10.35) | <0.001 | 6.76 (3.69–12.40) | <0.001 |
| Female sex |  |  | 0.96 (0.79–1.17) | 0.699 | 0.97 (0.78–1.21) | 0.802 | 1.06 (0.84–1.33) | 0.624 |
| SBP (mmHg) |  |  |  |  | 0.97 (0.97–0.98) | <0.001 | 0.98 (0.98–0.98) | <0.001 |
| Respiratory rate |  |  |  |  | 0.93 (0.90–0.97) | 0.002 | 0.94 (0.90–0.98) | 0.002 |
| Intubated in ED |  |  |  |  |  |  | 1.36 (1.00–1.84) | 0.050 |
| Care level: General ward |  |  |  |  |  |  | 0.49 (0.32–0.75) | <0.001 |
| Care level: Operating theatre |  |  |  |  |  |  | 0.45 (0.29–0.71) | <0.001 |
| Care level: Specialised ward |  |  |  |  |  |  | 0.56 (0.30–1.03) | 0.062 |
| Care level: ICU |  |  |  |  |  |  | 1.42 (0.94–2.15) | 0.100 |
| Time to procedure: 0-30 min |  |  |  |  |  |  | 2.58 (1.69–3.94) | <0.001 |
| Time to procedure: 31-60 min |  |  |  |  |  |  | 0.72 (0.38–1.36) | 0.305 |
| Time to procedure: 61-120 min |  |  |  |  |  |  | 1.08 (0.75–1.56) | 0.672 |
| Time to procedure: >120 min |  |  |  |  |  |  | 0.48 (0.34–0.70) | <0.001 |
| Time to CT: 0-20 min |  |  |  |  |  |  | 0.34 (0.23–0.50) | <0.001 |
| Time to CT: 21-30 min |  |  |  |  |  |  | 0.35 (0.24–0.50) | <0.001 |
| Time to CT: 31-60 min |  |  |  |  |  |  | 0.29 (0.20–0.41) | <0.001 |
| Time to CT: 61-120 min |  |  |  |  |  |  | 0.20 (0.12–0.31) | <0.001 |
| Time to CT: >120 min |  |  |  |  |  |  | 0.37 (0.24–0.56) | <0.001 |

## Additional file 3: Full logistic regression results: Unfavourable GOS

| Term | Unadjusted OR (95% CI) | Unadj. p | Patient char. OR (95% CI) | Patient char. p | Physiological OR (95% CI) | Physiological p | Care-process OR (95% CI) | Care-process p |
| --- | --- | --- | --- | --- | --- | --- | --- | --- |
| Cohort: blunt multisystem with TBI | 15.06 (11.99–18.90) | <0.001 | 19.83 (15.57–25.26) | <0.001 | 16.85 (13.16–21.58) | <0.001 | 7.80 (6.00–10.16) | <0.001 |
| Cohort: blunt multisystem without TBI | 8.80 (7.02–11.05) | <0.001 | 10.62 (8.33–13.54) | <0.001 | 8.71 (6.79–11.18) | <0.001 | 4.05 (3.09–5.31) | <0.001 |
| Cohort: Isolated severe TBI | 29.23 (18.80–45.44) | <0.001 | 30.29 (19.11–47.99) | <0.001 | 32.29 (20.26–51.48) | <0.001 | 10.86 (6.68–17.66) | <0.001 |
| Cohort: severe penetrating | 1.54 (1.26–1.90) | <0.001 | 3.07 (2.45–3.84) | <0.001 | 1.92 (1.50–2.45) | <0.001 | 0.87 (0.65–1.17) | 0.345 |
| Age (years) |  |  | 1.04 (1.03–1.04) | <0.001 | 1.04 (1.04–1.05) | <0.001 | 1.05 (1.04–1.05) | <0.001 |
| ASA: 2 |  |  | 1.26 (1.10–1.44) | 0.001 | 1.34 (1.17–1.54) | <0.001 | 1.21 (1.04–1.41) | 0.014 |
| ASA: 3 |  |  | 1.86 (1.57–2.19) | <0.001 | 1.87 (1.57–2.21) | <0.001 | 1.47 (1.22–1.78) | <0.001 |
| ASA: 4 |  |  | 2.85 (1.68–4.84) | <0.001 | 2.67 (1.56–4.57) | <0.001 | 2.81 (1.57–5.03) | <0.001 |
| Female sex |  |  | 1.00 (0.89–1.12) | 0.960 | 0.96 (0.85–1.09) | 0.555 | 1.21 (1.06–1.38) | 0.006 |
| SBP (mmHg) |  |  |  |  | 0.99 (0.98–0.99) | <0.001 | 0.99 (0.99–0.99) | <0.001 |
| Respiratory rate |  |  |  |  | 1.00 (0.98–1.02) | 0.713 | 0.98 (0.96–1.00) | 0.026 |
| Intubated in ED |  |  |  |  |  |  | 0.79 (0.62–1.01) | 0.058 |
| Care level: General ward |  |  |  |  |  |  | 1.67 (1.32–2.11) | <0.001 |
| Care level: Operating theatre |  |  |  |  |  |  | 5.28 (4.09–6.82) | <0.001 |
| Care level: Specialised ward |  |  |  |  |  |  | 7.42 (5.40–10.20) | <0.001 |
| Care level: ICU |  |  |  |  |  |  | 15.66 (11.91–20.58) | <0.001 |
| Time to procedure: 0-30 min |  |  |  |  |  |  | 1.23 (0.90–1.70) | 0.200 |
| Time to procedure: 31-60 min |  |  |  |  |  |  | 0.86 (0.59–1.25) | 0.428 |
| Time to procedure: 61-120 min |  |  |  |  |  |  | 1.05 (0.83–1.34) | 0.670 |
| Time to procedure: >120 min |  |  |  |  |  |  | 1.63 (1.33–2.00) | <0.001 |
| Time to CT: 0-20 min |  |  |  |  |  |  | 0.34 (0.27–0.43) | <0.001 |
| Time to CT: 21-30 min |  |  |  |  |  |  | 0.34 (0.27–0.43) | <0.001 |
| Time to CT: 31-60 min |  |  |  |  |  |  | 0.41 (0.33–0.51) | <0.001 |
| Time to CT: 61-120 min |  |  |  |  |  |  | 0.34 (0.26–0.44) | <0.001 |
| Time to CT: >120 min |  |  |  |  |  |  | 0.65 (0.51–0.85) | 0.001 |

## Additional file 4: Full logistic regression results: Opportunities for improvement

| Term | Unadjusted OR (95% CI) | Unadj. p | Patient char. OR (95% CI) | Patient char. p | Physiological OR (95% CI) | Physiological p | Care-process OR (95% CI) | Care-process p |
| --- | --- | --- | --- | --- | --- | --- | --- | --- |
| Cohort: blunt multisystem with TBI | 2.39 (1.80–3.19) | <0.001 | 2.31 (1.73–3.08) | <0.001 | 2.37 (1.77–3.17) | <0.001 | 1.78 (1.29–2.46) | <0.001 |
| Cohort: blunt multisystem without TBI | 4.11 (3.11–5.43) | <0.001 | 3.96 (3.00–5.24) | <0.001 | 4.03 (3.03–5.36) | <0.001 | 2.82 (2.07–3.83) | <0.001 |
| Cohort: Isolated severe TBI | 1.60 (0.98–2.62) | 0.062 | 1.45 (0.88–2.39) | 0.141 | 1.49 (0.91–2.46) | 0.115 | 1.08 (0.63–1.87) | 0.777 |
| Cohort: severe penetrating | 1.55 (1.05–2.28) | 0.028 | 1.64 (1.10–2.44) | 0.014 | 1.71 (1.14–2.56) | 0.009 | 1.40 (0.90–2.17) | 0.133 |
| Age (years) |  |  | 1.01 (1.00–1.01) | 0.037 | 1.01 (1.00–1.01) | 0.051 | 1.01 (1.00–1.01) | 0.049 |
| ASA: 2 |  |  | 1.08 (0.85–1.36) | 0.548 | 1.06 (0.84–1.34) | 0.625 | 0.95 (0.75–1.21) | 0.701 |
| ASA: 3 |  |  | 1.18 (0.88–1.58) | 0.267 | 1.17 (0.87–1.56) | 0.290 | 1.04 (0.77–1.40) | 0.786 |
| ASA: 4 |  |  | 0.79 (0.28–2.24) | 0.659 | 0.77 (0.27–2.19) | 0.629 | 0.68 (0.24–1.93) | 0.466 |
| Female sex |  |  | 0.85 (0.69–1.04) | 0.114 | 0.85 (0.69–1.05) | 0.135 | 0.90 (0.72–1.11) | 0.317 |
| SBP (mmHg) |  |  |  |  | 1.00 (1.00–1.00) | 0.451 | 1.00 (1.00–1.00) | 0.602 |
| Respiratory rate |  |  |  |  | 1.02 (1.00–1.04) | 0.019 | 1.01 (0.99–1.03) | 0.456 |
| Intubated in ED |  |  |  |  |  |  | 1.29 (0.94–1.77) | 0.111 |
| Care level: General ward |  |  |  |  |  |  | 3.05 (1.89–4.90) | <0.001 |
| Care level: Operating theatre |  |  |  |  |  |  | 4.14 (2.50–6.87) | <0.001 |
| Care level: Specialised ward |  |  |  |  |  |  | 6.95 (3.99–12.09) | <0.001 |
| Care level: ICU |  |  |  |  |  |  | 3.59 (2.12–6.06) | <0.001 |
| Time to procedure: 0-30 min |  |  |  |  |  |  | 0.87 (0.54–1.40) | 0.572 |
| Time to procedure: 31-60 min |  |  |  |  |  |  | 1.01 (0.54–1.89) | 0.966 |
| Time to procedure: 61-120 min |  |  |  |  |  |  | 1.79 (1.27–2.52) | <0.001 |
| Time to procedure: >120 min |  |  |  |  |  |  | 2.71 (2.07–3.57) | <0.001 |
| Time to CT: 0-20 min |  |  |  |  |  |  | 1.15 (0.76–1.75) | 0.502 |
| Time to CT: 21-30 min |  |  |  |  |  |  | 1.03 (0.69–1.54) | 0.894 |
| Time to CT: 31-60 min |  |  |  |  |  |  | 1.88 (1.29–2.75) | 0.001 |
| Time to CT: 61-120 min |  |  |  |  |  |  | 2.65 (1.76–3.99) | <0.001 |
| Time to CT: >120 min |  |  |  |  |  |  | 1.78 (1.15–2.74) | 0.009 |

## Additional file 5: Unadjusted and fully adjusted odds ratios by cohort

|  | **30-day mortality** | | **Unfavourable GOS** | | **OFI** | |
| --- | --- | --- | --- | --- | --- | --- |
| **Cohort** | **Mortality Unadj.** | **Mortality Adjusted** | **Unfav. GOS Unadj.** | **Unfav. GOS Adjusted** | **OFI Unadj.** | **OFI Adjusted** |
| Isolated severe TBI | 26.34 (19.94–34.79) | 21.54 (14.16–32.78) | 29.23 (18.80–45.44) | 10.86 (6.68–17.66) | 1.60 (0.98–2.62) | 1.08 (0.63–1.87) |
| Blunt multisystem with TBI | 9.50 (7.65–11.79) | 5.28 (3.89–7.17) | 15.06 (11.99–18.90) | 7.80 (6.00–10.16) | 2.39 (1.80–3.19) | 1.78 (1.29–2.46) |
| Penetrating truncal injury | 6.35 (4.88–8.26) | 2.83 (1.77–4.52) | 1.54 (1.26–1.90) | 0.87 (0.65–1.17) | 1.55 (1.05–2.28) | 1.40 (0.90–2.17) |
| Blunt multisystem without TBI | 4.45 (3.34–5.93) | 1.69 (1.13–2.52) | 8.80 (7.02–11.05) | 4.05 (3.09–5.31) | 4.11 (3.11–5.43) | 2.82 (2.07–3.83) |

## Additional file 6: Cause of death by TQIP cohort (multinomial logistic regression)

| **Cohort** | **Cause of death** | **Frequency** | **OR** | **95% CI** | **p-value** |
| --- | --- | --- | --- | --- | --- |
| Blunt multisystem without TBI (n=427) | Alive | 359 / 7,568 (4.7%) | — |  |  |
|  | TBI | 6 / 329 (1.8%) | 1.29 | 0.56, 2.98 | 0.547 |
|  | Bleeding | 28 / 131 (21.4%) | 44.98 | 22.21, 91.06 | **<0.001** |
|  | Multi-organ failure | 12 / 36 (33.3%) | 10.60 | 5.14, 21.85 | **<0.001** |
|  | Other | 22 / 183 (12.0%) | 3.21 | 2.02, 5.12 | **<0.001** |
| Blunt multisystem with TBI (n=587) | Alive | 417 / 7,568 (5.5%) | — |  |  |
|  | TBI | 109 / 329 (33.1%) | 20.23 | 14.94, 27.39 | **<0.001** |
|  | Bleeding | 21 / 131 (16.0%) | 29.04 | 13.91, 60.63 | **<0.001** |
|  | Multi-organ failure | 3 / 36 (8.3%) | 2.28 | 0.68, 7.71 | 0.184 |
|  | Other | 28 / 183 (15.3%) | 3.52 | 2.31, 5.37 | **<0.001** |
| Isolated severe TBI (n=246) | Alive | 115 / 7,568 (1.5%) | — |  |  |
|  | TBI | 119 / 329 (36.2%) | 80.08 | 57.20, 112.10 | **<0.001** |
|  | Bleeding | 0 | — |  |  |
|  | Multi-organ failure | 0 | — |  |  |
|  | Other | 7 / 183 (3.8%) | 3.19 | 1.46, 6.98 | **0.004** |
| Penetrating truncal injury (n=423) | Alive | 333 / 7,568 (4.4%) | — |  |  |
|  | TBI | 13 / 329 (4.0%) | 3.02 | 1.67, 5.48 | **<0.001** |
|  | Bleeding | 71 / 131 (54.2%) | 122.97 | 64.55, 234.27 | **<0.001** |
|  | Multi-organ failure | 1 / 36 (2.8%) | 0.95 | 0.13, 7.12 | 0.962 |
|  | Other | 5 / 183 (2.7%) | 0.79 | 0.32, 1.94 | 0.602 |
| Reference: Alive in the Non TQIP-cohort. Frequency: n / N (%), where N is the total count of that cause of death across all cohorts. Multinomial logistic regression. Deaths classified as Unknown excluded. | | | | | |

## Additional file 7: Individual GOS levels by TQIP cohort (multinomial logistic regression)

| **Cohort** | **GOS level** | **Frequency** |
| --- | --- | --- |
| Blunt multisystem without TBI (n=427) | Good recovery | 4 / 2,629 (0.2%) |
|  | Moderate disability | 101 / 2,773 (3.6%) |
|  | Severe disability | 253 / 2,225 (11.4%) |
|  | Dead/Vegetative | 69 / 671 (10.3%) |
| Blunt multisystem with TBI (n=587) | Good recovery | 1 / 2,629 (0.0%) |
|  | Moderate disability | 93 / 2,773 (3.4%) |
|  | Severe disability | 325 / 2,225 (14.6%) |
|  | Dead/Vegetative | 168 / 671 (25.0%) |
| Isolated severe TBI (n=246) | Good recovery | 3 / 2,629 (0.1%) |
|  | Moderate disability | 19 / 2,773 (0.7%) |
|  | Severe disability | 91 / 2,225 (4.1%) |
|  | Dead/Vegetative | 133 / 671 (19.8%) |
| Penetrating truncal injury (n=423) | Good recovery | 59 / 2,629 (2.2%) |
|  | Moderate disability | 216 / 2,773 (7.8%) |
|  | Severe disability | 57 / 2,225 (2.6%) |
|  | Dead/Vegetative | 91 / 671 (13.6%) |
| Distribution of GOS levels within each cohort. Frequency: n / N (%), where N is the total count of that GOS level across all cohorts. Odds ratios are not shown: the reference level (good recovery) is near-empty in the severe cohorts (n = 1-4), yielding unstable estimates. | | |

## Additional file 8: MICE convergence diagnostics

MICE convergence trace plots across 20 imputed datasets and 50 iterations. Each line represents the mean imputed value per iteration for one imputed dataset. Outcomes (survival, GOS, OFI) were included as predictors in the imputation model. Variables with very few missing values (marked with *) exhibit oscillating trace plots, which is expected; convergence for these was confirmed quantitatively (R-hat < 1.05).

## Additional file 9: OR trajectories across sequential models

Odds ratio trajectories across sequential regression models, for 30-day mortality, unfavourable GOS, and OFI by TQIP cohort.

*Points represent odds ratios with 95% confidence intervals. Dashed line indicates OR = 1 (reference). Patient characteristics: age, ASA, sex. Physiological: + SBP, respiratory rate. Care-process: + intubation, care level, time to procedure, time to CT (the time variables also encode whether the procedure/CT was performed).*

## Additional file 10: Reference cohort composition and sensitivity analyses

The non-TQIP reference cohort is large and clinically heterogeneous. To test whether the cohort odds ratios were sensitive to how it was defined, we describe the reference cohort (Table S10a), re-estimate the cohort odds ratios against two narrower reference groups—patients with NISS > 15 and patients with a full trauma-team (Trauma-1) activation (Table S10b)—and compare the four TQIP cohorts directly, with the reference removed (Table S10c).

**Table S10a. Baseline characteristics and outcomes of the non-TQIP reference cohort, stratified by injury-severity band and by trauma-team alert level (Trauma-1 vs Trauma-2/none).**

|  | **Overall** | **Injury severity (NISS)** | | | **Trauma team alert** | |
| --- | --- | --- | --- | --- | --- | --- |
|  | **N = 6,615^1^** | **NISS 1-8, N = 3,353 (51%)^1^** | **NISS 9-15, N = 1,413 (21%)^1^** | **NISS >15, N = 1,838 (28%)^1^** | **Trauma-1, N = 3,827 (58%)^1^** | **Trauma-2/none, N = 2,788 (42%)^1^** |
| **Age, years** | 42 (27, 61) | 36 (24, 53) | 47 (29, 65) | 52 (33, 69) | 41 (26, 60) | 44 (27, 62) |
| **Male sex** | 4,469 (68%) | 2,183 (65%) | 955 (68%) | 1,322 (72%) | 2,769 (72%) | 1,700 (61%) |
| **ASA ≥3** | 1,203 (18%) | 423 (13%) | 309 (22%) | 471 (26%) | 674 (18%) | 529 (19%) |
| **NISS** | 8 (3, 17) | 3 (1, 4) | 11 (9, 12) | 22 (17, 27) | 9 (3, 17) | 6 (2, 16) |
| **Intubated in ED** | 302 (4.6%) | 82 (2.4%) | 65 (4.6%) | 155 (8.4%) | 260 (6.8%) | 42 (1.5%) |
| **Respiratory rate** | 18 (16, 20) | 18 (16, 20) | 18 (16, 20) | 18 (16, 20) | 18 (16, 20) | 18 (16, 20) |
| **SBP, mmHg** | 136 (122, 150) | 136 (124, 150) | 137 (122, 153) | 137 (120, 151) | 137 (121, 150) | 136 (123, 150) |
| **GCS** | 15 (15, 15) | 15 (15, 15) | 15 (15, 15) | 15 (14, 15) | 15 (14, 15) | 15 (15, 15) |
| **Admission ward** |  |  |  |  |  |  |
| ED | 1,397 (21%) | 1,229 (37%) | 110 (7.8%) | 49 (2.7%) | 250 (6.5%) | 1,147 (41%) |
| General ward | 2,865 (43%) | 1,650 (49%) | 651 (46%) | 564 (31%) | 1,980 (52%) | 885 (32%) |
| Operating theatre | 1,222 (18%) | 266 (7.9%) | 462 (33%) | 493 (27%) | 806 (21%) | 416 (15%) |
| Specialised ward | 323 (4.9%) | 68 (2.0%) | 59 (4.2%) | 196 (11%) | 167 (4.4%) | 156 (5.6%) |
| ICU | 808 (12%) | 140 (4.2%) | 131 (9.3%) | 536 (29%) | 624 (16%) | 184 (6.6%) |
| **Trauma team activated** | 5,742 (87%) | 3,162 (94%) | 1,210 (86%) | 1,360 (74%) | 3,827 (100%) | 1,915 (69%) |
| **Emergency intervention** |  |  |  |  |  |  |
| No intervention | 5,309 (80%) | 3,098 (92%) | 1,068 (76%) | 1,133 (62%) | 2,887 (75%) | 2,422 (87%) |
| Thoracotomy | 13 (0.2%) | 1 (<0.1%) | 2 (0.1%) | 9 (0.5%) | 13 (0.3%) | 0 (0%) |
| Laparotomy | 85 (1.3%) | 46 (1.4%) | 17 (1.2%) | 22 (1.2%) | 80 (2.1%) | 5 (0.2%) |
| Pelvic packing | 2 (<0.1%) | 0 (0%) | 0 (0%) | 2 (0.1%) | 2 (<0.1%) | 0 (0%) |
| Revascularisation | 39 (0.6%) | 4 (0.1%) | 9 (0.6%) | 26 (1.4%) | 34 (0.9%) | 5 (0.2%) |
| Radiological intervention | 51 (0.8%) | 7 (0.2%) | 10 (0.7%) | 34 (1.8%) | 42 (1.1%) | 9 (0.3%) |
| Craniotomy | 168 (2.5%) | 0 (0%) | 3 (0.2%) | 165 (9.0%) | 79 (2.1%) | 89 (3.2%) |
| ICP monitoring only | 27 (0.4%) | 1 (<0.1%) | 2 (0.1%) | 24 (1.3%) | 20 (0.5%) | 7 (0.3%) |
| Other action§ | 921 (14%) | 196 (5.8%) | 302 (21%) | 423 (23%) | 670 (18%) | 251 (9.0%) |
| **Time to procedure, min** | 134 (79, 419) | 91 (69, 143) | 161 (94, 591) | 153 (85, 470) | 112 (73, 222) | 342 (136, 828) |
| **Time to CT, min** | 34 (21, 71) | 32 (20, 68) | 35 (22, 70) | 35 (22, 79) | 23 (18, 31) | 76 (51, 121) |
| **30-day mortality** | 270 (4.1%) | 49 (1.5%) | 49 (3.5%) | 164 (8.9%) | 198 (5.2%) | 72 (2.6%) |
| **30-day mortality excl. DOA** | 229 (3.5%) | 37 (1.1%) | 48 (3.4%) | 141 (7.7%) | 157 (4.1%) | 72 (2.6%) |
| **Cause of death** |  |  |  |  |  |  |
| TBI | 82 (35%) | 2 (5.6%) | 4 (9.1%) | 76 (50%) | 51 (29%) | 31 (52%) |
| Bleeding | 11 (4.7%) | 1 (2.8%) | 1 (2.3%) | 8 (5.2%) | 11 (6.3%) | 0 (0%) |
| Multi-organ failure | 20 (8.5%) | 2 (5.6%) | 5 (11%) | 13 (8.5%) | 16 (9.2%) | 4 (6.7%) |
| Other | 121 (52%) | 31 (86%) | 34 (77%) | 56 (37%) | 96 (55%) | 25 (42%) |
| **GOS at discharge** |  |  |  |  |  |  |
| Good recovery | 2,562 (39%) | 2,350 (70%) | 169 (12%) | 41 (2.2%) | 1,414 (37%) | 1,148 (41%) |
| Moderate disability | 2,344 (35%) | 765 (23%) | 838 (59%) | 741 (40%) | 1,379 (36%) | 965 (35%) |
| Severe disability | 1,499 (23%) | 205 (6.1%) | 375 (27%) | 918 (50%) | 867 (23%) | 632 (23%) |
| Dead/Vegetative | 210 (3.2%) | 33 (1.0%) | 31 (2.2%) | 138 (7.5%) | 167 (4.4%) | 43 (1.5%) |
| **OFI identified** | 311 (4.7%) | 57 (1.7%) | 72 (5.1%) | 182 (9.9%) | 183 (4.8%) | 128 (4.6%) |
| **OFI type** |  |  |  |  |  |  |
| Clinical judgement error | 100 (32%) | 15 (26%) | 17 (24%) | 68 (37%) | 43 (23%) | 57 (45%) |
| Delay in treatment | 46 (15%) | 10 (18%) | 11 (15%) | 25 (14%) | 37 (20%) | 9 (7.0%) |
| Inadequate protocols | 15 (4.8%) | 3 (5.3%) | 2 (2.8%) | 10 (5.5%) | 7 (3.8%) | 8 (6.3%) |
| Inadequate resources | 76 (24%) | 13 (23%) | 20 (28%) | 43 (24%) | 57 (31%) | 19 (15%) |
| Missed diagnosis | 53 (17%) | 15 (26%) | 18 (25%) | 20 (11%) | 28 (15%) | 25 (20%) |
| Potentially preventable deaths | 21 (6.8%) | 1 (1.8%) | 4 (5.6%) | 16 (8.8%) | 11 (6.0%) | 10 (7.8%) |
| ^1^Median (Q1, Q3); n (%) | | | | | | |
| Non-TQIP reference cohort only (n = 6615). Trauma-1 = full-team alert; Trauma-2/none = limited alert or no alert. Cause-of-death percentages are of deaths; OFI-type percentages are of OFIs; time-to-procedure and time-to-CT are medians among patients who underwent the procedure or CT. Missing values not shown. | | | | | | |

The reference cohort covers the full range of injury severity. The most severely injured patients in this group (NISS > 15) have an OFI rate of 9.9%, similar to several of the TQIP cohorts.

Against both narrower references (NISS > 15 and Trauma-1), the two highest-mortality cohorts (isolated severe TBI and blunt multisystem with TBI) retained elevated mortality odds ratios and penetrating truncal injury remained modestly elevated; the mortality/OFI dissociation was unchanged (Table S10b).

**Table S10b. Cohort odds ratios under the primary, most-severely-injured (NISS > 15), and Trauma-1 references.**

| **Outcome** | **Cohort** | **Model** | **Primary** | **NISS>15 ref.** | **Trauma-1 ref.** |
| --- | --- | --- | --- | --- | --- |
| 30-day mortality | Isolated severe TBI | Unadjusted | 26.34 (19.94–34.79) | 11.44 (8.50–15.40) | 20.54 (15.39–27.41) |
|  |  | Care-process adjusted | 21.54 (14.16–32.78) | 13.27 (8.63–20.40) | 17.56 (11.55–26.70) |
|  | Blunt multisystem with TBI | Unadjusted | 9.50 (7.65–11.79) | 4.13 (3.25–5.25) | 7.41 (5.89–9.32) |
|  |  | Care-process adjusted | 5.28 (3.89–7.17) | 3.26 (2.35–4.51) | 4.35 (3.17–5.98) |
|  | Blunt multisystem without TBI | Unadjusted | 4.45 (3.34–5.93) | 1.93 (1.43–2.62) | 3.47 (2.58–4.67) |
|  |  | Care-process adjusted | 1.69 (1.13–2.52) | 1.11 (0.73–1.68) | 1.36 (0.90–2.06) |
|  | Penetrating truncal injury | Unadjusted | 6.35 (4.88–8.26) | 2.76 (2.08–3.66) | 4.95 (3.77–6.51) |
|  |  | Care-process adjusted | 2.83 (1.77–4.52) | 1.64 (1.01–2.66) | 2.30 (1.41–3.74) |
| Unfavourable GOS | Isolated severe TBI | Unadjusted | 29.23 (18.80–45.44) | 7.54 (4.82–11.80) | 27.50 (17.65–42.86) |
|  |  | Care-process adjusted | 10.86 (6.68–17.66) | 3.96 (2.41–6.51) | 10.41 (6.41–16.91) |
|  | Blunt multisystem with TBI | Unadjusted | 15.06 (11.99–18.90) | 3.88 (3.06–4.93) | 14.17 (11.23–17.86) |
|  |  | Care-process adjusted | 7.80 (6.00–10.16) | 2.95 (2.24–3.89) | 7.32 (5.61–9.54) |
|  | Blunt multisystem without TBI | Unadjusted | 8.80 (7.02–11.05) | 2.27 (1.79–2.88) | 8.28 (6.57–10.44) |
|  |  | Care-process adjusted | 4.05 (3.09–5.31) | 1.71 (1.30–2.26) | 3.91 (2.98–5.14) |
|  | Penetrating truncal injury | Unadjusted | 1.54 (1.26–1.90) | 0.40 (0.32–0.50) | 1.45 (1.18–1.80) |
|  |  | Care-process adjusted | 0.87 (0.65–1.17) | 0.29 (0.21–0.40) | 0.79 (0.59–1.07) |
| OFI | Isolated severe TBI | Unadjusted | 1.60 (0.98–2.62) | 0.72 (0.43–1.19) | 1.57 (0.95–2.60) |
|  |  | Care-process adjusted | 1.08 (0.63–1.87) | 0.75 (0.43–1.30) | 1.15 (0.66–2.02) |
|  | Blunt multisystem with TBI | Unadjusted | 2.39 (1.80–3.19) | 1.07 (0.79–1.46) | 2.35 (1.74–3.18) |
|  |  | Care-process adjusted | 1.78 (1.29–2.46) | 1.22 (0.87–1.69) | 1.82 (1.29–2.55) |
|  | Blunt multisystem without TBI | Unadjusted | 4.11 (3.11–5.43) | 1.85 (1.37–2.48) | 4.04 (3.01–5.42) |
|  |  | Care-process adjusted | 2.82 (2.07–3.83) | 1.97 (1.43–2.70) | 2.74 (1.97–3.80) |
|  | Penetrating truncal injury | Unadjusted | 1.55 (1.05–2.28) | 0.69 (0.46–1.04) | 1.52 (1.02–2.27) |
|  |  | Care-process adjusted | 1.40 (0.90–2.17) | 0.90 (0.57–1.42) | 1.30 (0.83–2.04) |
| All cells are odds ratios (95% CI). Reference: non-TQIP cohort. Columns give the primary reference and two restricted references (most severely injured, NISS > 15; and full-team, Trauma-1), all four TQIP cohorts unchanged. Care-process adjusted = full model (patient characteristics, physiological status, care processes). | | | | | |

With the reference removed and the four TQIP cohorts compared directly (n = 1,683; reference: blunt multisystem without TBI), the mortality gradient and its inversion for OFI were reproduced (Table S10c).

**Table S10c. Between-cohort odds ratios with the non-TQIP reference excluded (reference: blunt multisystem without TBI).**

| **Outcome** | **Cohort** | **Unadjusted** | **Care-process adjusted** |
| --- | --- | --- | --- |
| 30-day mortality | Isolated severe TBI | 5.92 (4.13–8.49) | 14.85 (8.60–25.63) |
|  | Blunt multisystem with TBI | 2.13 (1.56–2.92) | 3.39 (2.18–5.27) |
|  | Penetrating truncal injury | 1.43 (1.01–2.02) | 1.22 (0.69–2.15) |
| Unfavourable GOS | Isolated severe TBI | 3.32 (2.03–5.42) | 2.55 (1.43–4.54) |
|  | Blunt multisystem with TBI | 1.71 (1.25–2.34) | 1.72 (1.20–2.46) |
|  | Penetrating truncal injury | 0.18 (0.13–0.24) | 0.12 (0.08–0.19) |
| OFI | Isolated severe TBI | 0.39 (0.23–0.67) | 0.44 (0.24–0.82) |
|  | Blunt multisystem with TBI | 0.58 (0.40–0.84) | 0.64 (0.43–0.95) |
|  | Penetrating truncal injury | 0.38 (0.24–0.59) | 0.42 (0.25–0.70) |
| All cells are odds ratios (95% CI). Reference: blunt multisystem without TBI (the lowest-mortality TQIP cohort). The non-TQIP reference is excluded entirely; models are fitted on the four TQIP cohorts only (n = 1,683). Care-process adjusted = full model (patient characteristics, physiological status, care processes). The unadjusted contrasts are identical to re-levelling the primary unadjusted model; the adjusted contrasts are an independent refit on the four cohorts and therefore differ. | | | |

## Additional file 11: National trauma team activation criteria

Patients enter the trauma registry through trauma team activation or, without activation, an Injury Severity Score > 9. Activation follows the national Swedish criteria (*Nationella traumalarmskriterier*, Säker Traumavård): physiological and anatomical criteria trigger a full (Level 1) trauma team, and mechanism-of-injury criteria a limited (Level 2) team.

**Table S11. National Swedish trauma team activation criteria (adult).**

| **Criterion group (team level)** | **Criterion** |
| --- | --- |
| **Physiological — Level 1 (full team)** | Need for ventilatory support |
|  | Respiratory rate <10 or >29 /min |
|  | Systolic blood pressure <90 mmHg or non-palpable radial pulse |
|  | Reaction Level Scale >3 or Glasgow Coma Scale <13 |
| **Anatomical — Level 1 (full team)** | Penetrating injury to the neck, head, torso, or an extremity proximal to the elbow/knee |
|  | Open or depressed skull fracture |
|  | Facial or neck injury with a threatened airway |
|  | Unstable or deformed chest |
|  | Severe pelvic pain or suspected pelvic fracture |
|  | Suspected spinal cord injury |
|  | More than two long-bone fractures |
|  | Amputation proximal to the hand/foot |
|  | Major external haemorrhage |
|  | Burns >18% of body surface area or inhalation injury |
| **Mechanism of injury — Level 2 (limited team)** | Motor-vehicle collision >50 km/h without a seatbelt |
|  | Ejection from a vehicle |
|  | Entrapment with extrication time >20 min |
|  | Motorcycle (or equivalent) collision >35 km/h |
|  | Fall >5 m |
| **Observanda (not activation criteria — prompt physician contact)** | Progressive deterioration of a suspected seriously injured patient |
|  | Increased bleeding risk (anticoagulants) |
|  | Age <5 or >60 years |
|  | Serious pre-existing illness |
|  | Hypothermia <35 °C |
|  | Under the influence of drugs |
|  | Pregnancy |
| Adult criteria (Säker Traumavård); paediatric thresholds for patients <15 years are omitted as these patients were excluded. | |

*Source:* [*Nationella traumalarmskriterier, Säker Traumavård (Löf), 2024 update*](https://wp.lof.se/wp-content/uploads/Nationella-traumalarmskriterier-2024.pdf)*. Translated and abridged for the adult trauma population.*
